# Gut microbes combined with metabolomics reveal the protective effects of Qijia Rougan decoction against CCl_4_-induced hepatic fibrosis

**DOI:** 10.3389/fphar.2024.1347120

**Published:** 2024-03-28

**Authors:** Xue Li, Xinyi Xu, Sian Tao, Yue Su, Li Wen, Dong Wang, Jibin Liu, Quansheng Feng

**Affiliations:** School of Basic Medicine, Chengdu University of Traditional Chinese Medicine, Chengdu, China

**Keywords:** Qijia Rougan decoction, hepatic fibrosis, carbon tetrachloride, gut microbiota, metabolomics

## Abstract

**Background:** The occurrence and development of Hepatic fibrosis (HF) are closely related to the gut microbial composition and alterations in host metabolism. Qijia Rougan decoction (QJ) is a traditional Chinese medicine compound utilized clinically for the treatment of HF with remarkable clinical efficacy. However, its effect on the gut microbiota and metabolite alterations is unknown. Therefore, our objective was to examine the impact of QJ on the gut microbiota and metabolism in Carbon tetrachloride (CCl_4_)-induced HF.

**Methods:** 40% CCl_4_ was used to induce HF, followed by QJ administration for 6 weeks. Serum biochemical analyses, histopathology, immunohistochemistry, RT-PCR, 16S rRNA gene sequencing, and non-targeted metabolomics techniques were employed in this study to investigate the interventional effects of QJ on a CCl_4_-induced HF model in rats.

**Results:** This study demonstrated that QJ could effectively ameliorate CCl_4_-induced hepatic inflammation and fibrosis. Moreover, QJ upregulated the expression of intestinal tight junction proteins (TJPs) and notably altered the abundance of some gut microbes, for example, 10 genera closely associated with HF-related indicators and TJPs. In addition, metabolomics found 37 key metabolites responded to QJ treatment and strongly associated with HF-related indices and TJPs. Furthermore, a tight relation between 10 genera and 37 metabolites was found post correlation analysis. Among them, *Turicibacter*, *Faecalibaculum*, *Prevotellaceae UCG 001*, and *unclassified Peptococcaceae* may serve as the core gut microbes of QJ that inhibit HF.

**Conclusion:** These results suggest that QJ ameliorates hepatic inflammation and fibrosis, which may be achieved by improving intestinal tight junctions and modulating gut microbiota composition as well as modulating host metabolism.

## 1 Introduction

Hepatic fibrosis (HF) is a dynamic and highly integrated process involving molecules, cells and tissues that drives progressive overaccumulation of extracellular matrix (ECM) components and is maintained by activation of myofibroblasts (Parola and Pinzani 2019; Kisseleva and Brenner 2021). Viral hepatitis and alcoholic and non-alcoholic fatty liver disease are the most common causes of HF, while progressive non-alcoholic fatty liver disease (NAFLD) is becoming the primary cause of terminal liver disease worldwide (Devarbhavi et al., 2023; Huang et al., 2023). HF is a crucial stage of Chronic liver diseases (CLDs) that may advance to cirrhosis and eventually to hepatocellular carcinoma (HCC). The reversibility of HF is supported by evidence from preclinical studies, clinical trials and clinical observations (Hammel et al., 2001; Mazzola et al., 2017; Mauro et al., 2018; Nakano et al., 2020; Boyer-Diaz et al., 2021; Yoshino et al., 2021; Xing et al., 2023). Effective antifibrotic therapy is highly beneficial, even in patients with end-stage HF (Parola and Pinzani 2019). However, to date, there has been no drug approved by FDA for HF treatment in clinical practice. Consequently, there is a pressing medical need for antifibrotic therapies to hinder the progression of CLDs and the development of HCC.

Integrated analyses of gut microbes and metabolomics are increasingly used to study the complex mechanisms of HF. The gut and liver maintain a closely interconnected communication system facilitated by the systemic circulation, portal vein, and biliary tract (Tripathi et al., 2018). Enteric barrier impairment is a crucial factor in the transmission of hazardous germs and their metabolites to the liver. It typically does not induce liver damage alone but exacerbates hepatocellular injury and inflammation and further aggravates pre-existing liver disease (Albillos et al., 2020; Zhao X. J. et al., 2021; Tilg et al., 2022; Hammerich and Tacke, 2023; Han et al., 2023). Studies on alterations in gut microbes and metabolites in HF have also been reported previously (Wei et al., 2016; Caussy et al., 2018; Bajaj et al., 2020; Lee et al., 2020; Cox et al., 2022). Numerous studies have demonstrated that botanical drugs and their extracts are capable of suppressing HF by restoring enteric barriers and modulating intestinal dysbacteriosis and metabolites (Zhao J. et al., 2021; Hu et al., 2021; Fu et al., 2022; Liu et al., 2022; Yue et al., 2022; Zheng et al., 2022; Chang et al., 2023; Guo et al., 2023; Hu et al., 2023). Studies have suggested that probiotics such as *Lactobacillus GG* (Bajaj et al., 2014), *Bacteroides dorei* (Park et al., 2023), *Clostridium butyricum* and *Bifidobacterium longum infantis* (Lu et al., 2023), as well as gut microbial metabolites such as indole-3-propionic acid (Sehgal et al., 2021; Yuan et al., 2023) and Trimethylamine N-oxide (Zhou D. et al., 2022; Nian et al., 2023), are beneficial in HF.

Qijia Rougan Decoction (QJ) is modified from the Sanjia San prescription in the “*Wenyilun*” (Youke, 2003) and includes *Astragali radix* [*Fabaceae*; *Astragalus mongholicus Bunge*] 30 g, *Angelicae sinensis radix* [*Apiaceae*; *Angelica sinensis (Oliv.) Diels*] 9 g, *Trionycis carapax* [*Trionychidae*; *Trionyx sinensis Wiegmann*] 15 g, *Eupolyphaga steleophaga* [*Corydiidae*; *Eupolyphaga sinensis*] 12 g, *Salviae miltiorrhizae radix et rhizoma* [*Lamiaceae*; *Salvia miltiorrhiza Bunge*] 18 g, *Carthami flos* [*Asteraceae*; *Carthamus tinctorius L.*] 18 g, *Persicae semen* [*Rosaceae*; *Prunus persica (L.) Batsch*] 18 g, *Sparganii rhizome* [Typhaceae; *Sparganium stoloniferum*] 15 g, *Curcumae rhizome* [*Zingiberaceae*; *Curcuma phaeocaulis Valeton*] 15 g, and *Glycyrrhizae radix et rhizome* [*Fabaceae*; *Glycyrrhiza uralensis Fisch.*] 6 g. According to the traditional theory, the main effects of QJ are replenishing qi and activating blood circulation, dissipating stagnation and unblocking collaterals. High-performance liquid chromatography (HPLC) tandem mass spectrometry (MS) was performed to determine the chemical components of QJ. The identified components with mzCloud best-match scores >95 were ursolic acid, tanshinone IIA, isoliquiritigenin, formononetin, 18-β-glycyrrhetinic acid, cryptotanshinone, daidzein, and nicotinic acid, additionally, QJ inhibits CCl_4_-induced HF through the TGF-β signaling pathway (Chen et al., 2022). Ursolic acid can alleviate HF by inhibiting the NOX4/NLRP3 (Nie et al., 2021) and NOX2/NLRP3 (Wan et al., 2022) signaling pathways. Ursolic acid can also improve the flora imbalance in HF mice and protect the intestinal mucosal barrier in HF rats (Wan et al., 2019; Zhang et al., 2019). Tanshinone IIA can alleviate HF by promoting the proliferation and differentiation of endogenous liver stem cells (Yang et al., 2020) and inhibiting the TGF-β1/Smad signaling pathway (Xu et al., 2022). Isoliquiritigenin plays an anti-fibrotic role *in vivo* and *in vitro* through caveolin-1-mediated iron death of hepatic stellate cells (HSCs) (Huang et al., 2022). The chemical components 18-β-glycyrrhetinic acid and cryptotanshinone can improve HF by promoting apoptosis of HSCs. Another study carried out proteomics analysis and verified that QJ inhibits hepatocyte death and the Akt/mTOR pathway (Chen et al., 2023). Moreover, QJ has been found to ameliorate ECM deposition in HF by regulating the JAK1/STAT6-microRNA-23a feedback loop in macrophage M2 polarization (Zheng et al., 2023). However, the effects of QJ on HF and its mechanisms remain to be explored, especially regarding regulation of gut microbes and their metabolites.

In this study, we used 16S rRNA gene sequencing and non-targeted metabolomics to investigate the role of QJ on intestinal microbes and metabolites in CCl_4_-induced HF rats, attempting to lay a more theoretical foundation for prospective utilization.

## 2 Methods

### 2.1 Preparation of Qijia Rougan decoction

The drugs of QJ were purchased from Sichuan New Green Pharmaceutical Technology Development Co., Ltd. The preparation method was described in detail in previous study (Chen, et al., 2022) and is briefly described as follows: according to the national pharmacopoeia standard, the drugs were soaked, and 1.5 L of pure water was added and the mixture brought to a boil. The drug solution was then filtered, and the above steps were repeated. Finally, the two drug solutions were mixed, concentrated, and stored in the refrigerator for further use. A patient needs 156 g/d crude drugs per 70 kg body weight. The rat needs 14 g/kg/d crude drugs following the method from *Experimental Course of Pharmacology of Traditional Chinese Medicine* (Xinguo, 2017). The high dose (QJ) is twice the medium dose. In this research, a high dose of QJ (28 g/kg) was chosen as the optimal dose in previous study (Chen, et al., 2022) with no significant side effects.

### 2.2 Animal experimentation and treatment

Twenty-four male Sprague-Dawley rats (RRID: MGI: 5651135) at 6–8 weeks, weighing approximately 160–180 g, were purchased from Beijing SPF Biotechnology Co., Ltd. with License No. SCXK (Beijing) 2019-0008. Rats were maintained in a room on a 12-h light/dark cycle with 50%–60% humidity and a temperature of 20°C–24°C. Free access to both water and food was provided. Animal experiments were approved by the Ethics Committee for Animal Experiments of Chengdu University of Traditional Chinese Medicine (No. 2021-66) and performed in accordance with the National Institutes of Health guidelines. The rats were randomly divided into three groups (*n* = 8 per group): the control (C) group—an equal amount of olive oil solution and saline during modeling and gavage; the model (M) group—CCl_4_ olive oil solution (40%, 3 mL/kg, s.c.) twice a week for 8 weeks, then once per week for 6 weeks and an equal amount of saline daily for the same 6 weeks; the QJ group—CCl_4_ olive oil solution twice a week for 8 weeks, then once per week for 6 weeks and QJ daily for the same 6 weeks. Rats were anesthetized with 3% sodium pentobarbital (50 mg/kg, i.p.). The contents of the large intestine were collected under sterile conditions in a clean bench, and then samples of abdominal aortic blood, liver, and ileum were collected. Rats were finally euthanized by an overdose of sodium pentobarbital.

### 2.3 Serum biochemical measurement

Blood samples were centrifuged at 3500 rpm for 10 min. Serum was collected and analyzed for rat aspartate aminotransferase (AST), alanine aminotransferase (ALT), and alkaline phosphatase (ALP) using a fully automated blood biochemistry analyzer (Mindray BS-240 VET, China).

### 2.4 Histopathology analysis

The liver and ileum were fixed with 4% paraformaldehyde for 24 h and then trimmed, dehydrated, embedded, and sectioned (4 μm). Hematoxylin-eosin (HE) staining was performed on both liver and ileum tissues, whereas only liver tissues underwent Masson staining. Stained sections were transformed into images through the use of a pathology scanner (HS6, Sunny Optical Technology, China). The study quantitatively measured the positive areas of Masson staining utilizing Image-Pro Plus 6.0 (RRID:SCR 007369). Each sample was measured by randomly selecting four fields of view and calculating the mean integrated optical density (IOD) values.

### 2.5 Immunohistochemistry analysis

Immunohistochemistry (IHC) was performed according to Qu et al. (2023). Paraffin-embedded tissue sections were deparaffinized with xylene and rehydrated in an ethanol gradient. Sodium citrate solution was used for antigen retrieval in microwave oven, and 3% H_2_O_2_ solution was utilized for endogenous peroxidase blocking. Nonspecific antigens were blocked with goat serum. Afterwards, the slices were incubated overnight at 4°C with Collagen I (1:500, Abcam Cat# ab270993, RRID: AB 2927551), α-SMA (1:100, Cell Signaling Technology Cat# 19245, RRID: AB 2734735), Claudin-1 (1:150, Thermo Fisher Scientific Cat# 51-9000, RRID:AB 2533916), Occludin (1:150, Thermo Fisher Scientific Cat# 40-4700, RRID:AB 2533468) and ZO-1 (1:150, Thermo Fisher Scientific Cat# 61-7300, RRID:AB 2533938).Secondary antibodies were incubated for 1 h, followed by DAB chromogenization and hematoxylin staining. The sections were subsequently dehydrated and fixed. Semi-quantitative analysis of the images was performed utilizing Image-Pro Plus, and mean IOD values were calculated by randomly selecting four fields of view for each section.

### 2.6 Real-time quantitative PCR analysis

Total RNA was isolated from liver and ileum tissues via FastPure Tissue Total RNA Isolation Kit (RC112, Vazyme, China), and then cDNA was synthesized by reverse transcription using HiScript III All-in-one RT SuperMix (R333, Vazyme, China). Finally, qPCR measurements were accomplished with a fluorescence quantitative PCR instrument (qTOWER3G, Jena, Germany) using Taq Pro Universal SYBR qPCR Master Mix (Q712, Vazyme, China), and relative gene expression was calculated by 2^−ΔΔCT^ method. The gene primer sequences are detailed in Table 1.

**TABLE 1:** The primers used in this study.

Gene	Forward primer (5′-3′)	Reverse primer (5′-3′)
IL-1β	GAC​TTC​ACC​ATG​GAA​CCC​GT	CAG​GGA​GGG​AAA​CAC​ACG​TT
IL-6	CAG​AGG​ATA​CCA​CCC​ACA​ACA​GA	GAA​CTC​CAG​AAG​ACC​AGA​GCA​GA
TNF-α	CCC​TGG​TAT​GAG​CCC​ATG​TA	CGG​ACT​CCG​TGA​TGT​CTA​AGT​A
Col1a1	TCA​CTG​CAA​GAA​CAG​CGT​AG	AAG​CGT​GCT​GTA​GGT​GAA​TC
α-SMA	GGA​TCA​GCG​CCT​TCA​GTT​CT	AGG​GCT​AGA​AGG​GTA​GCA​CA
Occludin	TTC​TTT​CCT​TAG​GCG​ACC​G	GCC​TGT​AAG​GAG​GTG​GAC​TC
Claudin-1	GAC​TGT​GGA​TGT​CCT​GCG​TT	CAT​CAA​GGC​TCT​GGT​TGC​CT
ZO-1	ACC​TTG​AGC​AGC​CAC​CAT​AC	CGA​GTT​GGG​TAG​GGC​TGT​TT
GAPDH	GCC​TTC​TCT​TGT​GAC​AAA​GT	CTT​GCC​GTG​GGT​AGA​GTC​ATA

### 2.7 High-throughput 16S rRNA gene sequencing and data analysis

TGuide S96 Magnetic Soil/Stool DNA Kit (Tiangen Biotech Co., Ltd., China) was used to extract genomic DNA from intestinal contents following the manufacturer’s instructions. The V1-V9 hypervariable regions of the 16S rRNA gene were amplified using primers (27F: 5′-AGRGTTTGATYNTGGCTCAG -3′; 1492R: 5′-TASGGHTACCTTGTTASGACTT -3′). Amplicon concentrations were quantified and equimolarly normalized before pooling for sequencing on the PacBio Sequel II platform (Beijing Biomarker Technologies Co., Ltd., China).

USEARCH (version 10.0) was used to assign sequences that exceeded the 97% similarity threshold to an operational taxonomic unit (OTU). Taxonomy annotation of the OTUs was performed based on the Naive Bayes classifier in QIIME2 (RRID:SCR 021258) using the SILVA database (RRID:SCR 006423) with a confidence threshold of 70%. Alpha analysis was conducted using QIIME2 to characterize the biodiversity complexity of each sample. Beta diversity calculations were analyzed by principal coordinate analysis (PCoA) to assess the diversity in samples for species complexity. Linear discriminant analysis (LDA) coupled with effect size (LEfSe) was applied to evaluate the differentially abundant taxa.

### 2.8 Non-targeted metabolomics

The LC/MS system consisted of a UPLC (Acquity I-Class PLUS, Waters, United States) in tandem with a high-resolution MS (Xevo G2-XS QTOF, Waters, United States) on an Acquity UPLC HSS T3 column (1.8 μm 2.1*100 mm, Waters, United States) operated in positive/negative ion mode with mobile phase A: 0.1% formic acid aqueous solution and mobile phase B: 0.1% formic acid acetonitrile. MSe mode acquisition of primary and secondary mass spectral data were under MassLynx V4.2 (RRID:SCR 014271) control. ESI ion source parameters were capillary voltage set to 2000 V for positive ion mode or −1500 V for negative ion mode, cone voltage at 30 V, ion source temperature maintained at 150°C, desolvent gas temperature kept at 500°C, backflush gas flow rate of 50 L/h, and desolventizing gas flow rate of 800 L/h.

The raw data were processed using Progenesis QI software to extract and align peaks, in addition to conducting various data processing operations. This was achieved by using the METLIN database (RRID:SCR 010500) and Biomark’s self-created library for identification. Furthermore, theoretical fragment identification and mass deviation were both limited to 100 ppm. Following the normalization of the original peak area information with the total peak area, subsequent analysis is carried out. The identified metabolites were searched for classification and pathway information in KEGG (RRID:SCR 012773), HMDB (RRID:SCR 007712), and LIPID MAPS (RRID:SCR 006579) databases. Based on the grouping, fold change (FC) was calculated, and a t-test was used to calculate the P value for each metabolite. The orthogonal partial least squares discriminant analysis (OPLS-DA) model was utilized to screen the differential metabolites. The screening criteria were FC >1, P-value < 0.05 and VIP >1.5. Differential metabolites of KEGG pathway enrichment significance were calculated using the hypergeometric distribution test.

### 2.9 Statistical analysis

SPSS 26.0 (RRID:SCR 002865) was utilized for statistical analysis, and most bar charts were created using GraphPad Prism (RRID:SCR 002798). Experimental data were presented as mean ± standard deviation (SD). Group comparisons were executed through either t-test or one-way ANOVA, and multiple comparisons were performed using the Bonferroni method (equal variances) or Tamhane T2 (unequal variances). We conducted Spearman correlation analysis using the R language (RRID:SCR 001905) and plotted heat maps. Statistical significance was set at *p* < 0.05.

## 3 Results

### 3.1 QJ ameliorated CCl_4_-induced liver injury

**FIGURE 1:**
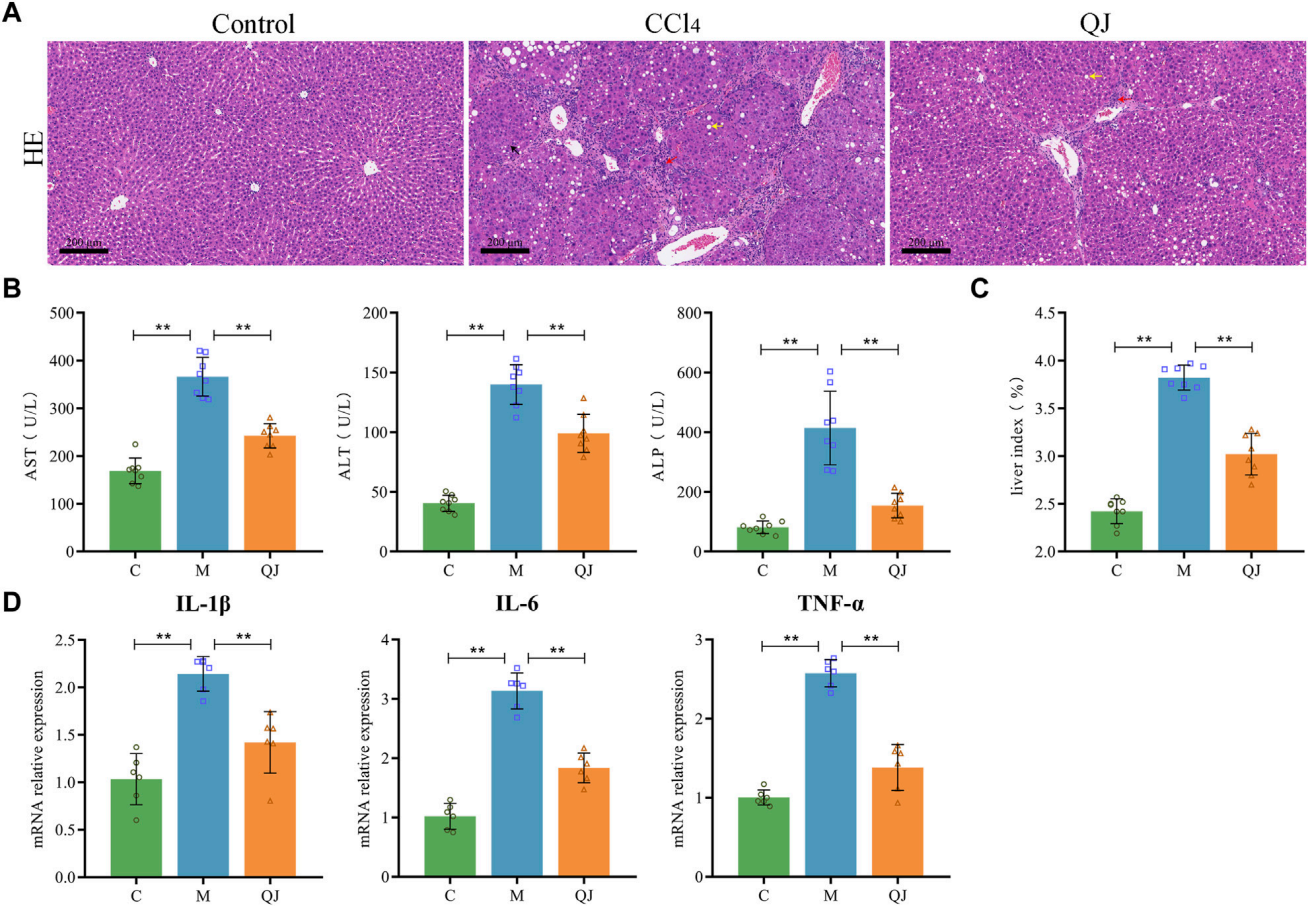
QJ ameliorated CCl_4_-induced liver injury. **(A)** H&E staining of liver sections, scale bar = 200 μm; Steatosis (yellow arrow), inflammatory cell infiltration (red arrow), and necrosis (black arrow). **(B)** Liver function indices: AST, ALT, and ALP (*n* = 8). **(C)** Calculation of liver index: Liver weight/Body weight × 100% (*n* = 8). **(D)** mRNA relative expression of IL-1β, IL-6, TNF-α in liver (*n* = 6). *, *p* <0.05. **, *p* <0.01.

Liver function tests displayed a significant increase in serum levels of AST, ALT and ALP in the M group compared with the C group. After QJ treatment, these levels were significantly reduced (Figure 1B). QJ also significantly downregulated the liver index in HF (Figure 1C). As illustrated in Figure 1A, HE staining revealed that hepatocytes in group C were normal in shape and tightly arranged, without any discernible pathological or inflammatory cellular infiltration in the liver parenchyma. After CCl_4_ treatment, hepatocytes exhibited obvious signs of steatosis, inflammation, and necrosis, all of which were alleviated by QJ. The levels of hepatic inflammatory indices in each group were detected by qPCR. The results showed that IL-1β, IL-6, and TNF-α levels were observably increased in the M group, and QJ treatment significantly downregulated this change, which further confirmed that QJ inhibited CCl_4_-induced liver inflammation (Figure 1D).

### 3.2 QJ inhibited ECM production

**FIGURE 2:**
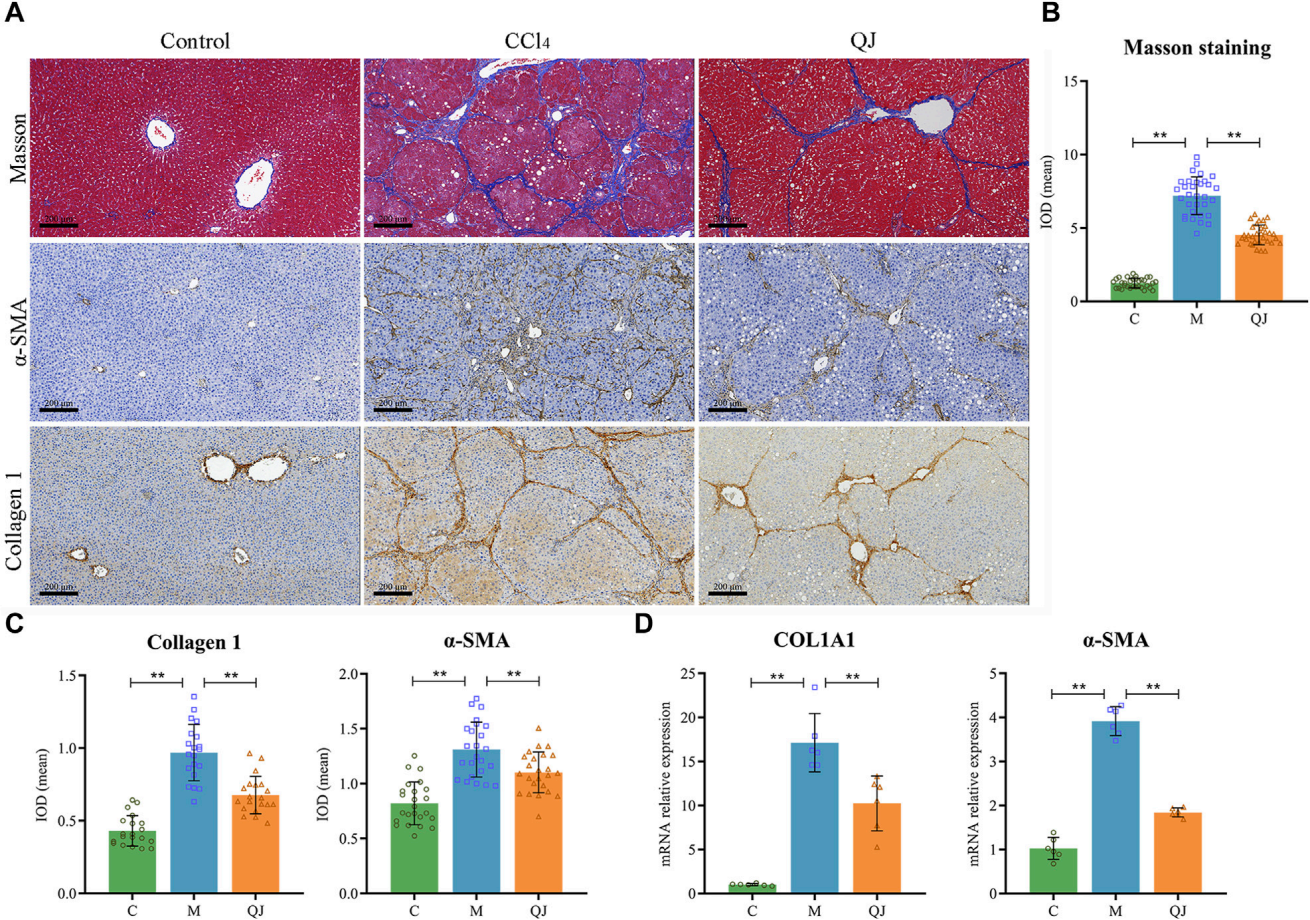
QJ attenuates CCl_4_-induced hepatic fibrosis. **(A)** Masson staining and IHC staining of Collagen 1 and α-SMA for liver sections, scale bar = 200 µm. **(B)** Semi-quantitative analysis of Masson staining (*n* = 8). **(C)** The mean IOD of Collagen 1 (*n* = 5) and α-SMA (*n* = 6) in IHC staining. **(D)** mRNA relative expression of COL1A1 and α-SMA in the liver (*n* = 6). *, *p* <0.05; **, *p* <0.01.

Masson staining and its semi-quantitative analysis (Figures 2A, B) showed that massive fibrous streaks and even pseudolobule formation were visible in group M with respect to group C, while QJ treatment significantly attenuated CCl_4_-induced fibrosis. Histologically, HF was characterized by disruption of hepatic architecture and excessive deposition of ECM, with type I collagen being a predominant constituent of ECM. HSC activation is the major cellular source of ECM production, and α-SMA is a marker of HSC activation. Collagen I and α-SMA expression in liver of each group was determined using IHC and qPCR. The results indicate that the mean IOD values and mRNA expression of Collagen I and α-SMA significantly increased in the M group but were significantly inhibited after treatment with QJ (Figures 2A, C, D). The above results demonstrated the inhibitory impact of QJ on CCl_4_-induced HF.

### 3.3 QJ ameliorated intestinal injury and upregulated the expression of tight junction proteins

**FIGURE 3:**
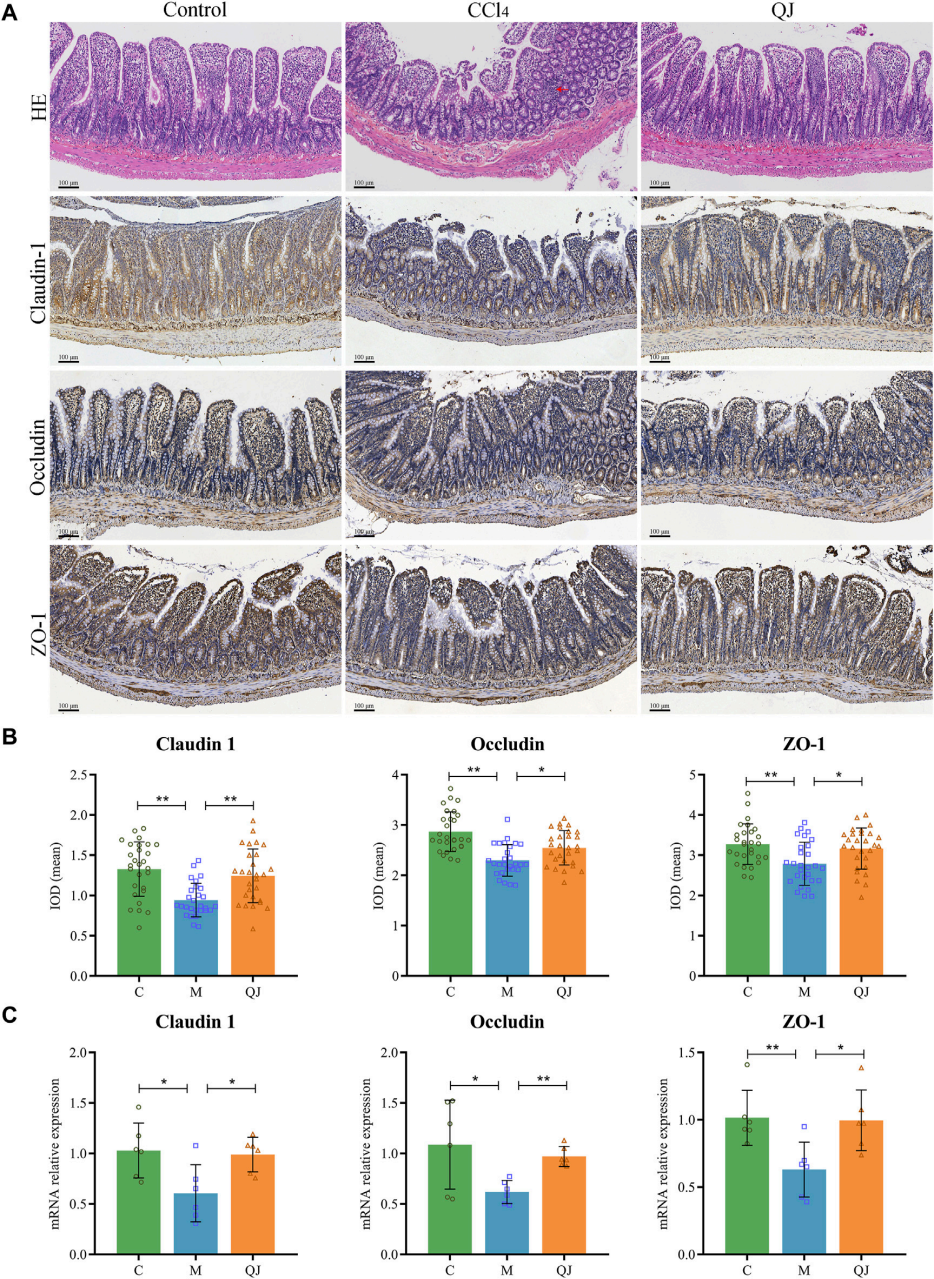
QJ ameliorated intestinal injury and upregulated expression of tight junction proteins. **(A)** HE staining and IHC staining of Claudin-1, Occludin and ZO-1 for ileum sections, inflammatory cell infiltration (red arrow). **(B)** The mean IOD of Claudin 1, Occludin and ZO-1 in IHC staining (*n* = 7). **(C)** mRNA relative expression of Claudin-1, Occludin and ZO-1 in ileum (*n* = 6). *, *p* <0.05; **, *p* <0.01.

HE staining of the ileum showed clear intestinal tissue structure, normal cell morphology, and no obvious inflammatory changes in the C group (Figure 3A). In contrast, CCl_4_ modeling resulted in sparsely arranged connective tissue, reduced intestinal villus height and crypt depth, and the infiltration of inflammatory cells. However, QJ administration restored intestinal villus height and crypt depth without observable inflammatory changes.

Tight junctions are vital for maintaining the structural integrity and normal function of the enteric epithelial barrier by serving as the primary connection between intestinal mucosal epithelial cells. Disruption of tight junctions can damage the intestinal epithelial barrier, which may allow intestinal microbes and their metabolites to translocate and exacerbate pre-existing liver disease (Wang and Liu, 2021). Research has shown that the expression of Occludin in the small intestine of mice decreased significantly after the first injection of CCl_4_, and the bacterial translocation (CCl_4_, 4 times) preceded the imbalance of gut microbes (CCl_4_, 24 times; bridging fibrosis) (Fouts et al., 2012). The increased permeability caused by the change of intestinal tight junction may explain the early bacterial translocation in the CCl_4_-induced liver injury model. Claudin-1, Occludin and ZO-1 are important components of intestinal tight junction proteins (TJPs). Their mean IOD values and mRNA expression were measured by IHC and qPCR. IHC analysis of the ileum, depicted in Figures 3A, B, displayed a significant reduction in the mean IOD values for Claudin-1, Occludin, and ZO-1 after CCl_4_ modeling. Nevertheless, their values were restored post QJ administration. Moreover, the mRNA expression measured by qPCR was consistent with the IHC results (Figure 3C). The findings indicated that CCl_4_ modeling causes downregulation of TJPs, which may lead to harmful gut microbes and their metabolites affecting the liver through the gut-liver axis. However, QJ has a beneficial effect on CCl_4_-induced intestinal epithelial barrier damage.

### 3.4 QJ modulated the structure and composition of the gut microbiota

To investigate the impact of QJ on the composition of gut microbiota in HF rats, we collected intestinal contents and subjected them to high-throughput 16S rRNA gene sequencing. Venn diagrams demonstrated the number of shared and unique OTUs between groups (Supplementary Figure S1A). The flattening rarefaction curve indicates sufficient sequencing depth across samples (Supplementary Figure S1B). The flattening of rank abundance curve reveals that the samples contained a rich and homogeneous species composition (Supplementary Figure S1C).

**FIGURE 4:**
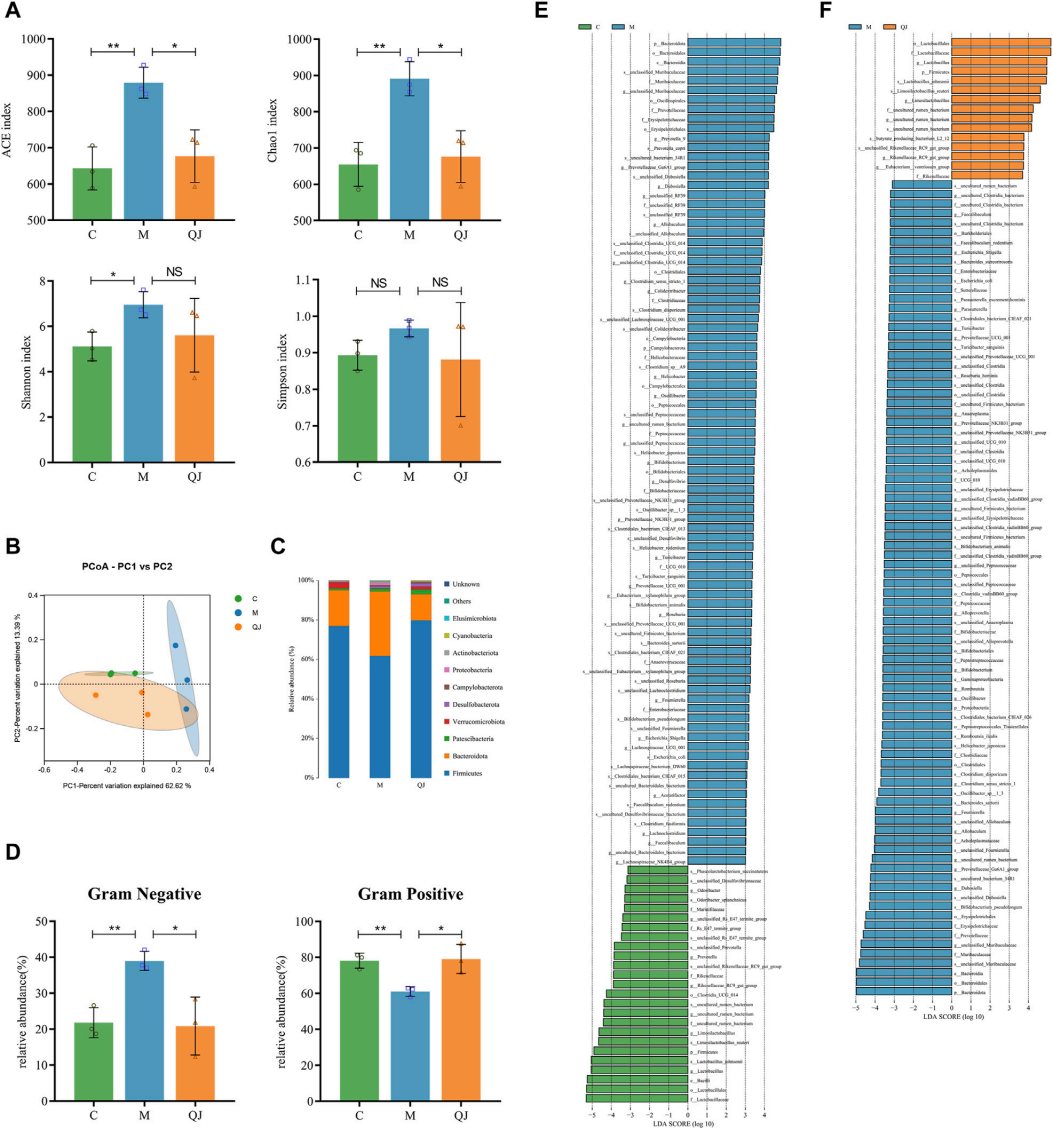
Effect of QJ on the structure and composition of gut microbiota (*n* = 3). **(A)** Alpha diversity analysis. **(B)** PCoA analysis. **(C)** Changes in gut microbiota composition at the phylum level. **(D)** Gut microbiota phenotype based on BugBase, including Gram-positive, and Gram-negative. **(E)** Differential gut microbes between C and M groups by LEfSe analysis. **(F)** Differential gut microbes between M and QJ groups by LEfSe analysis. The threshold of the logarithmic score of LDA analysis was 3.0. The color of the bar chart indicates that gut microbe corresponds to the group with a higher abundance. NS, *p* >0.05; *, *p* <0.05; **, *p* <0.01.

The richness and diversity of gut microbiota were assessed using alpha diversity. The indices of Ace, Chao 1, and Shannon demonstrated that the richness and diversity of the gut microbiota increased following CCl_4_ induction. However, after the QJ administration, Ace and Chao 1 indices decreased (Figure 4A). The results indicated that QJ treatment counteracted the effects of HF on the richness of gut microbiota.

Then, weighted unifrac-based PCoA and ANOSIM were used to analyze the changes in the structure of the gut microbial community among the groups. ANOSIM (R=0.613, *p* = 0.016, Supplementary Figure S1D) showed that there were significant differences in gut microbial structure among the three groups. PCoA (PC1 = 62.62%, PCo2 = 13.39%) showed that the C group was completely separated from the M group. Moreover, the gut microbial composition of the QJ group was closer to the C group than to the M group (Figure 4B). The above results indicate that QJ regulated the community structure of the rat’s gut microbiota.

Bugbase is used to predict the potential phenotypes in each group in which the abundances of gram-negative and positive bacteria changed significantly (Figure 4D). Compared with group C, group M significantly raised the abundance of Gram-negative bacteria and significantly reduced that of Gram-positive bacteria, while QJ restored it. At the phylum level, we identified 10 gut microbes in total, of which *Firmicutes* and *Bacteroidota* were the two most dominant phyla, making up over 90% of the gut microbial composition (Figure 4C). As shown in Figures 4C–E, the M group exhibited reduced levels of beneficial bacteria *Firmicutes* and a higher presence of *Bacteroidota* than the C group. Following QJ treatment, however, the relative abundances of both *Firmicutes* and *Bacteroidetes* were reversed (Figures 4C, F).

LEfSe analysis was conducted to identify statistically significant biomarkers and the dominant gut microbes in each group (Figures 4E, F; Supplementary Table S1, S2). The larger the logarithmic LDA score, the more significant differences of that gut microbe between groups. Based on an LDA score >3, the M group displayed an enrichment of *Muribaculaceae* and *Prevotellaceae* in *Bacteroidota*, as well as *Erysipelotrichaceae*, *Clostridiaceae*, and *Peptococcaceae* in *Firmicutes, Enterobacteriaceae* in *Proteobacteria,* and *Bifidobacteriaceae* in *Actinobacteriota*. Both the C and QJ groups demonstrated enrichment of *Lactobacillaceae*, *uncultured rumen bacterium* (belonging to *Clostridia UCG 014*), *Rikenellaceae*, etc. Research results indicated that gut microbial structure underwent considerable alterations after induction by CCl_4_, and QJ was capable of adjusting or improving these alterations. As illustrated in Figures 4E, F, marked alterations in the abundance of 19 shared genera were recognized at the genus level. Furthermore, at the species level, QJ treatment resulted in downregulation of *Escherichia coli* and upregulation of *Lactobacillus johnsonii* and *Limosilactobacillus reuteri*, i.e., *Lactobacillus reuteri*. These gut microbes may potentially have an essential role in QJ inhibition of HF.

### 3.5 QJ improved metabolic disturbances in CCl_4_-induced hepatic fibrosis

**FIGURE 5:**
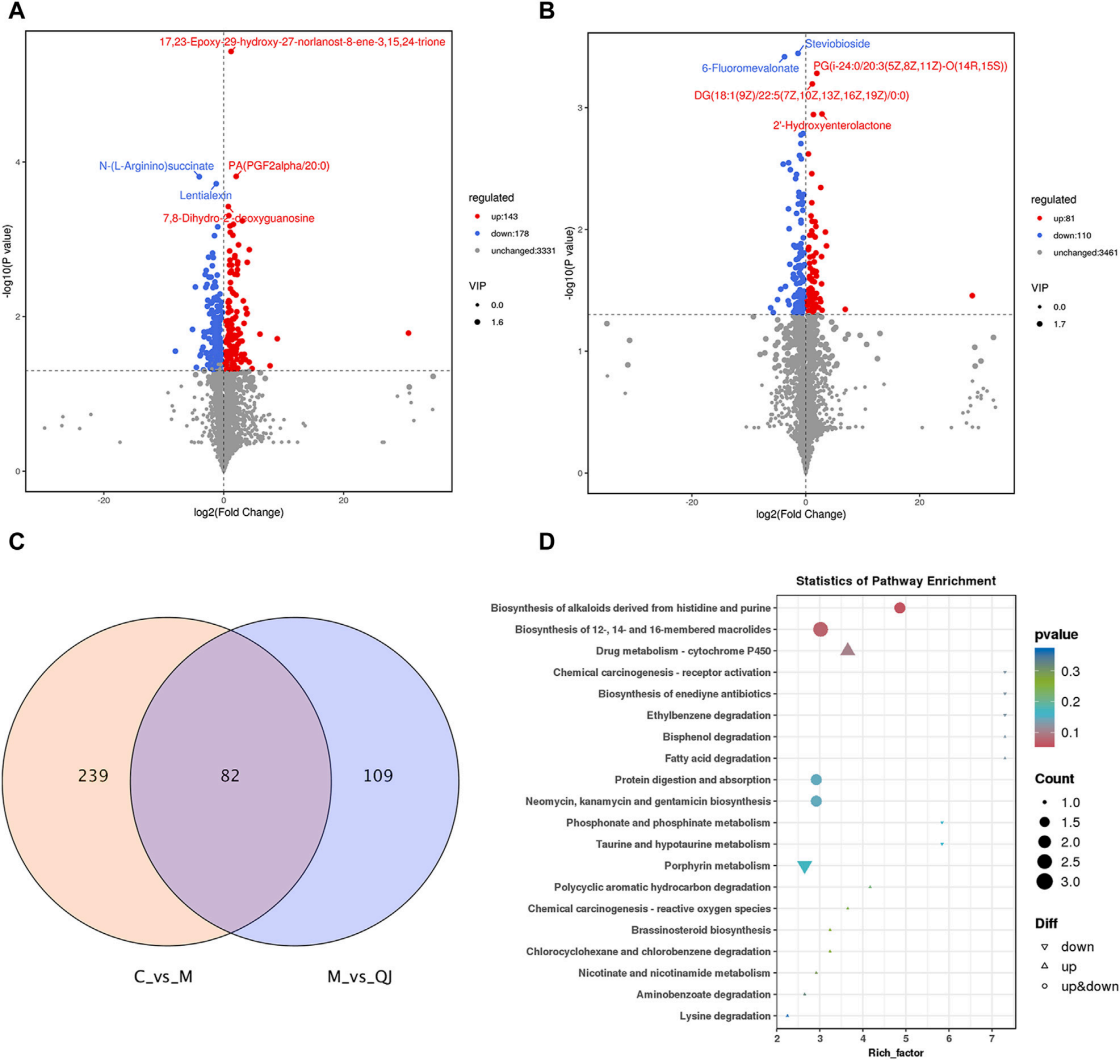
QJ improved metabolic disturbances in CCl_4_-induced hepatic fibrosis (*n* = 3). **(A)** Volcano plot between C and M groups. **(B)** Volcano plot between M and QJ groups. **(C)** Venn diagram of differential metabolites. **(D)** Visual analysis of enrichment pathway of altered metabolites between M and QJ groups.

To investigate the metabolic characteristics of each group, non-targeted metabolomics analysis was conducted using intestinal content samples. OPLS-DA showed that the C group was separated from the M group, and the M group was separated from the QJ group (Supplementary Figure S2A, D). The corresponding permutation tests established good validity and reliability, showing no "overfitting” (Supplementary Figure S2B, E). Volcano plots display the differential metabolites (Figures 5A, B). The metabolic pathways of QJ in inhibiting HF were analyzed based on the differential metabolites between the M and QJ groups. Relevant KEGG pathways included Drug metabolism—cytochrome P450, Fatty acid degradation, Taurine and hypotaurine metabolism, and Lysine degradation (Figure 5D).

Between C vs. M and M vs. QJ, there were 82 common differential metabolites (co-differential metabolites), all of which were inversely regulated by QJ (Figure 5C). After excluding metabolites that lacked annotation in KEGG or LIPID MAPS or HMDB databases, as well as metabolites that were not categorized in HMDB, there were 61 co-differential metabolites (Supplementary Table S3), which may be important metabolites for QJ inhibition of HF.

### 3.6 Spearman correlation analysis

Spearman correlation analysis was utilized to calculate the associations between 19 shared genera or 61 co-differential metabolites and indices related to HF (AST, ALT, ALP, liver index, and mRNA expression of IL-1β, IL-6, TNF-α, COL1a1, and α-SMA), as well as mRNA expression of TJPs (Claudin-1, Occludin, and ZO-1).

**FIGURE 6:**
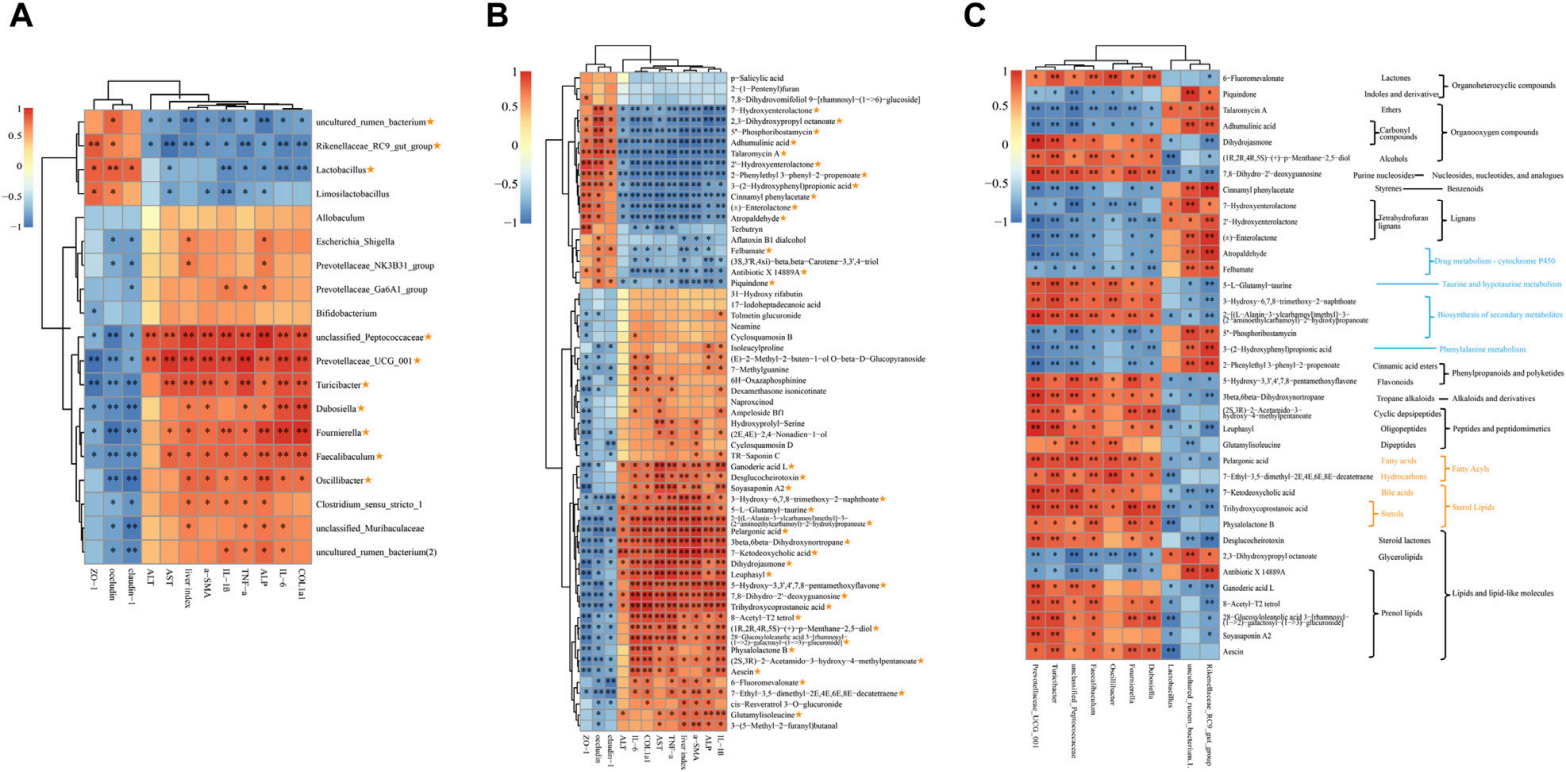
Spearman correlation analysis. **(A)** Correlations analysis between HF-related indices, TJPs and 19 shared genera. **(B)** Correlations analysis between HF-related indices, TJPs and 61 co-differential metabolites. **(C)** Correlations analysis between 10 shared genera and 36 key metabolites. The metabolic pathway or classification was presented on the right: the blue notes are KEGG metabolic pathways; the yellow notes are the LIPID MAPS classification; the black notes are HMDB classification. Yellow asterisk: (1) ≥8 indices were intimately correlated with certain genus or metabolite; (2) reversed by QJ treatment. *, *p* <0.05; **, *p* <0.01.

Figure 6A indicates that 10 of the 19 shared genera display a strong association with both HF-related indices and TJPs. Specifically, *uncultured rumen bacterium*, *Rikenellaceae RC9 gut group*, and *Lactobacillus* exhibit a significant negative correlation with HF-related indices, while demonstrating a significant positive correlation with TJPs. Conversely, *unclassified Peptococcaceae*, *Prevotellaceae UCG 001*, *Turicibacter*, *Dubosiella*, *Fournierella*, *Faecalibaculum*, and *Oscillibacter* exhibit the opposite relationship.

There were 37 metabolites out of 61 co-differential metabolites intimately correlated with HF-related indices and TJPs and reversed by QJ (Figure 6B), which may be the key metabolites for QJ to regulate metabolic changes. Among these 37 metabolites, 7-Hydroxyenterolactone, 2,3-Dihydroxypropyl octanoate, 5″-Phosphoribostamycin, Adhumulinic acid, Talaromycin A, 2′-Hydroxyenterolactone, 2-Phenylethyl 3-phenyl-2-propenoate, 3-(2-Hydroxyphenyl) propionic acid, Cinnamyl phenylacetate, (±)-Enterolactone, Atropaldehyde, Felbamate, Antibiotic X 14889A, and Piquindone showed significant negative correlations with HF-related indices and significant positive correlations with TJPs, while the other metabolites showed positive correlations with HF-related indices and negative correlations with TJPs. In addition, Figure 6C shows that these metabolites are involved in lignans, bile acids, Lipids, Drug metabolism - cytochrome P450, Phenylalanine metabolism, and Taurine and hypotaurine metabolism, etc.

Figure 6C illustrates the correlation between the aforementioned 10 genera and 37 differential metabolites. Piquindone, Talaromycin A, Adhumulinic acid, Cinnamyl phenylacetate, 7-Hydroxyenterolactone, 2′- Hydroxyenterolactone, (±)-Enterolactone, Atropaldehyde, Felbamate, 5″-Phosphoribostamycin, 3-(2- Hydroxyphenyl)propionic acid, 2-Phenylethyl 3-phenyl-2-propenoate, 2,3 Dihydroxypropyl octanoate, and Antibiotic X 14889A were positively correlated with *Rikenellaceae RC9 gut group*, *uncultured rumen bacterium*, and *Lactobacillus*, and adversely related to *Prevotellaceae UCG 001*, *Turicibacter*, *unclassified Peptococcaceae*, *Faecalibaculum*, *Oscillibacter*, *Fournierella, and Dubosiella*; the opposite was true for the other metabolites. *Turicibacter*, *Faecalibaculum*, *Prevotellaceae UCG 001*, and *unclassified Peptococcaceae* were the most highly correlated with 37 differential metabolites among the 10 genera. Notably, both *Faecalibaculum* and *Turicibacter* were significantly correlated with all 37 differential metabolites, while *Prevotellaceae UCG 001* and *unclassified Peptococcaceae* were significantly correlated with 36 metabolites. These four genera might serve as the core gut microbes that inhibit HF by QJ.

## 4 Discussion

**FIGURE 7:**
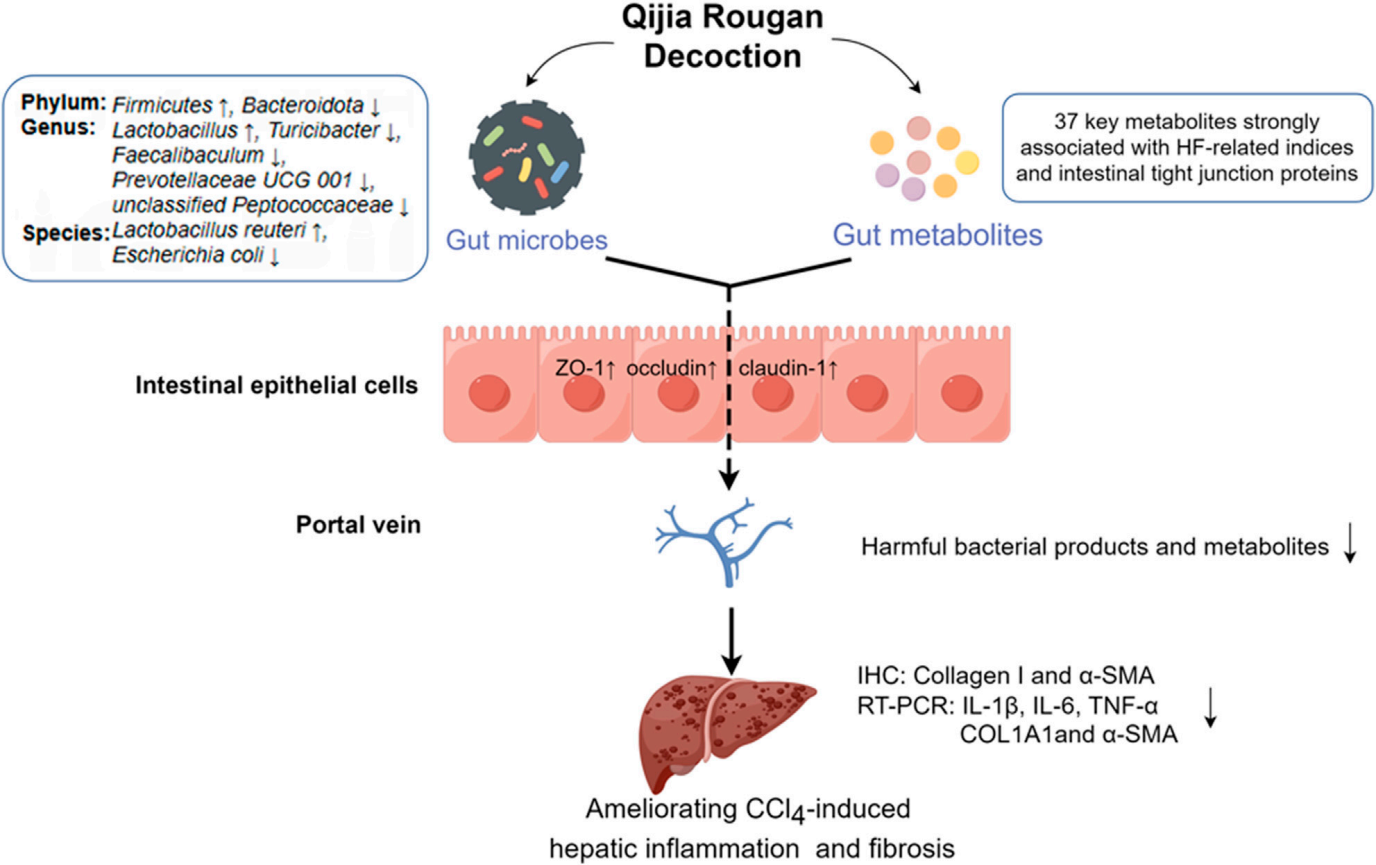
QJ may exert anti-HF effects by modulating intestinal dysbacteriosis and metabolic disorders as well as improving intestinal tight junction. By Figdraw (https://www.figdraw.com).

Hepatic fibrosis imposes a high global burden of disease, yet there is a huge unmet medical need for anti-fibrotic therapies. However, traditional Chinese medicine has great potential in anti-HF. In this study, we found that QJ may exert anti-HF effects by modulating intestinal dysbacteriosis and metabolic disorders (Figure 7).

The previous results of animal experiment demonstrated that the high-dose QJ group has the best effect of anti-HF (Chen, et al., 2022). Therefore, only the high-dose QJ group was used in this study following the 4R rules. In this study, QJ improved liver function and suppressed liver inflammation and fibrosis, which was consistent with previous studies (Chen, et al., 2022; Chen, et al., 2023; Zheng, et al., 2023). The study revealed that the intervention with QJ reinstated the expression of TJPs (Claudin-1, Occludin, and ZO-1) in rats with HF so as to preserve the normal function of the intestinal barrier, and diminished liver injury via regulation of harmful microbes and metabolites in intestine. Intestinal barrier impairment caused by TJPs deficiency has been reported as a major contributor to the pathomechanisms of clinical cirrhosis and HF induced by choline-deficient L-amino acid-defined diet, thioacetamide, common bile duct ligation and CCl_4_ (Assimakopoulos et al., 2012; Lee et al., 2021; Liu et al., 2021; Enomoto et al., 2022).

The result of 16S rRNA sequencing reveals that QJ regulated richness and community structure of the gut microbiota. The richness of gut microbes in HF was remarkably increased, consistent with previous research results (Chen et al., 2016; Zheng, et al., 2022). Moreover, QJ reversed CCl_4_-induced alterations in the abundance of some gut microbes in HF. At the phylum level, in group M, the abundance of *Firmicutes* decreased, while that of *Bacteroides* increased, which was similar to the previously reported intestinal dysbacteriosis in HF (Schwimmer et al., 2019; Han et al., 2021; Li et al., 2021). However, QJ reversed these changes. A prospective cohort study found that negativicutes are enriched, and *Clostridia* is significantly reduced in NAFLD-cirrhosis probands (Oh et al., 2020). *Clostridia UCG 014* exhibits an inverse correlation with ALT and AST levels in acute hepatic failure experiments, indicating its potentially beneficial effect on liver health (Zhao et al., 2022). In this study, the abundance of gram-negative bacteria significantly increased, and that of beneficial bacteria *uncultured rumen bacterium* (belonging to *Clostridia UCG 014, Clostridia*) decreased post CCl_4_ induction. Lipopolysaccharide (LPS) is a component of the cell wall of Gram-negative bacteria. Gram-negative bacteria, which were more abundant in the M group, might produce more LPS. In gut-liver axis, the damage of the intestinal barrier promotes the translocation of LPS from intestine to liver, and LPS can aggravate liver injury by activating the TLR4 signaling pathway. Furthermore, this research also found that QJ increased the abundance of other gut microbes that may be protective against liver injury or HF, such as *Lactobacillus*, *Lactobacillus johnsonii*, *Lactobacillus reuteri*, and *Rikenellaceae*. *Lactobacillus* is essential for maintaining intestinal homeostasis. One study reported that *Lactobacillus johnsonii JNU3402* protects against NAFLD via lactate PKA-SREBP-1c pathway (Hong et al., 2023). *Lactobacillus* consumption may be a viable measure to prevent and treat HF, as suggested by previous research (Santos et al., 2020; Won et al., 2023). *Lactobacillus reuteri* may alleviate alcohol-induced liver injury by enhancing the FXR signaling pathway (Cheng et al., 2022). Additionally, *Lactobacillus reuteri* can mediate intestinal TJPs expression through the Nrf-2/HO-1-NF-κB pathway, leading to improved intestinal barrier function in rats with acute liver failure (Zhou Q. et al., 2022). Moreover, decreased *Rikenellaceae* counts are observed in cirrhotic patients with small intestinal bacterial overgrowth (Maslennikov et al., 2022).

However, the abundance of *Erysipelotrichaceae* (*Turicibacter*, *Faecalibaculum*, and *Dubosiella*), *Enterobacteriaceae* (*Escherichia Shigella*, and *E. coli*), *Fournierella*, and *Prevotellaceae UCG 001* were the highest in group M, comprising potential harmful bacteria. QJ downregulated these bacteria. *Erysipelotrichaceae* (*Turicibacter* or *Faecalibaculum*) is increased in NAFLD mice (Somm et al., 2021; Xiong et al., 2021). NAFLD patients with markedly fibrosis show an increased abundance of *Turicibacter* compared to NAFLD with no/mild HF (Rodriguez-Diaz et al., 2022). *Erysipelotrichaceae* levels are three times higher in cirrhotic patients with HCC versus those without HCC (Piñero et al., 2019). In addition, a significant increase in *Enterobacteriaceae* abundance is found in patients with HF, and translocated *E. coli* in the liver could exacerbate HF in NAFLD mice by inducing endothelial-mesenchymal transition via the TLR5/MYD88/TWIST1 pathway (Champion et al., 2023; Shen et al., 2023). Lanthier et al. discovered that *Escherichia Shigella* characterizes the gut microbiota of MAFLD fibrotic subjects (Lanthier et al., 2021). Meanwhile, Bentong ginger oleoresin displays potent hepatoprotective and intestinal flora-modulating effects in NAFLD mice, thereby reducing the abundance of *Fournierella* (Wang K. et al., 2022). Increased *Prevotellaceae UCG 001* is found in D-galactosamine-induced liver injury (Li et al., 2019) and acute alcoholic liver injury (Yi et al., 2021).

At the genus level, QJ upregulated the abundance of *uncultured rumen bacterium*, *Rikenellaceae RC9 gut group* and *Lactobacillus*, and downregulated *unclassified Peptococcaceae*, *Prevotellaceae UCG 001*, *Turicibacter*, *Dubosiella*, *Fournierella*, *Faecalibaculum,* and *Oscillibacter*. Moreover, correlation analysis showed that the above 10 intestinal microbes were closely associated with HF-related indices and TJPs. Therefore, we concluded that they are key gut microbes associated with QJ inhibition of CCl_4_-induced HF.

Many studies have reported that traditional Chinese medicine and their bioactive components, such as curcumol (Zheng, et al., 2022) and phyllanthus emblica aqueous extract (Luo et al., 2022), ameliorate HF by modulating metabolic changes. In addition, Geniposide, tormentic acid, Ganlong capsules, and phillygenin have been found to alleviate CCl_4_-induced HF by regulating glycerophospholipid, amino acid, arachidonic acid, or bile acids metabolism (Yang et al., 2021; Wang C. et al., 2022; Lin et al., 2022; Lv et al., 2023). Thus, this study also examined the effect of QJ on intestinal content metabolism by untargeted metabolomics and identified 61 important co-differential metabolites that could be inversely regulated by QJ. Of those, 37 metabolites showed strong associations with HF-related indices and TJPs, suggesting that they may serve as key markers in the regulation of QJ on metabolism. These metabolites are involved in lignans, bile acids, Lipids, Drug metabolism - cytochrome P450, Phenylalanine metabolism, and Taurine and hypotaurine metabolism, etc.

The metabolites 2′-Hydroxyenterolactone, 7-Hydroxyenterolactone, and (±)-Enterolactone belong to lignans. In this study, they showed a significant negative correlation with HF-related indices and a significant positive correlation with TJPs. Lignans in *Bupleurum marginatum Wall.ex DC* (Liu et al., 2018), Forsythiae fructus (Wang et al., 2021; Wang C. et al., 2023) and Schisandra chinensis (Wang H.-Q. et al., 2022; Wang R. et al., 2023) have been reported to exert anti-HF effects. Within this study, two bile acid-related metabolites exhibited significant positive correlations with HF-related indices and notable negative correlations with TJPs. Trihydroxycoprostanoic acid (i.e., 3α, 7α, and 12α-trihydroxy-5β-cholestanoic) serves as an intermediate in the biosynthesis of cholesterol to bile acids (Kawai et al., 2022). 7-ketodeoxycholic acid belongs to the deoxycholic acids. It is upregulated in NASH mice (Miao et al., 2023) and increases with NAFLD disease progression and fibrosis stage (Smirnova et al., 2022). The metabolites 2-Phenylethyl 3-phenyl-2-propenoate and 5-Hydroxy-3,3′,4′,7,8-Pentamethoxyflavone are members of the phenylpropanoid. Phenylpropanoid metabolism commences with the amino acid phenylalanine (Yu and Jez, 2008). The metabolite 3-(2-Hydroxyphenyl) propionic acid was significantly negatively correlated with HF-related indices and positively correlated with TJPs, and it was involved in phenylalanine metabolism. A study reported that in children with HF after biliary atresia, significantly decreased phenylalanine metabolism is detected by the (13) C-phenylalanine breath test (Wada et al., 2007). The metabolite 5-L-Glutamyl-taurineis, an intermediate in Taurine and hypotaurine metabolism, was significantly and positively correlated with all HF-related indices. Meanwhile, its increase is associated with hyperlipidemia (Zeng et al., 2020), and aurantio-obtusin-induced hepatotoxicity (Xu et al., 2020). The study carried out by Luo, Xiaomin et al. showed that the therapeutic efficacy of Phyllanthus emblica aqueous extract on HF in NAFLD may be related to Taurine and hypotaurine metabolism (Luo, et al., 2022).

Furthermore, we also revealed the correlation between 10 genera and 37 co-differential metabolites that were closely associated with HF-related indices, TJPs, and regulated by QJ. Among them, *Turicibacter*, *Faecalibaculum*, *Prevotellaceae UCG 001*, and *unclassified Peptococcaceae* were the genera most closely associated with the 37 co-differential metabolites, which may serve as the core gut microbes for QJ inhibition of HF.

Nonetheless, our current study has a few limitations including a small sample size of 16s rRNA sequencing and non-targeted metabolomics, and the fact that only correlation studies were performed. The specific mechanism of action should be further clarified in future research. In addition, the modulation of gut microbes by QJ needs to be verified by expanding the sample size and fecal microbe transplantation in germ-free mice, and the mechanism of key metabolites on HF should be verified using an *in vitro* model. Also, further studies are needed to confirm the mechanism of linkage between gut microbiota and host metabolic alterations, and to explore specific gut microbes and related metabolites as important targets of QJ inhibition of HF.

## 5 Conclusion

In summary, our study demonstrated that QJ suppresses CCl_4_-induced hepatic inflammation and fibrosis. For the first time, we suggest that these effects may be related to the modulation of gut microbes and metabolic disorders as well as the improvement of intestinal tight junction.

## Data Availability

The datasets presented in this study can be found in online repositories. The names of the repository/repositories and accession number(s) can be found below: https://www.ncbi.nlm.nih.gov/, PRJNA1046280.
