# Supplementary material for: Gut microbes combined with metabolomics reveal the protective effects of Qijia Rougan decoction against CCl4-induced hepatic fibrosis
Source: Front Pharmacol. 2024 Mar 28;15:1347120. doi: 10.3389/fphar.2024.1347120 (PMC11007057; doi:10.3389/fphar.2024.1347120)
Supplement: Supplementary file 2 [file DataSheet1.pdf]

Supplementary Material

1 Supplementary Figure

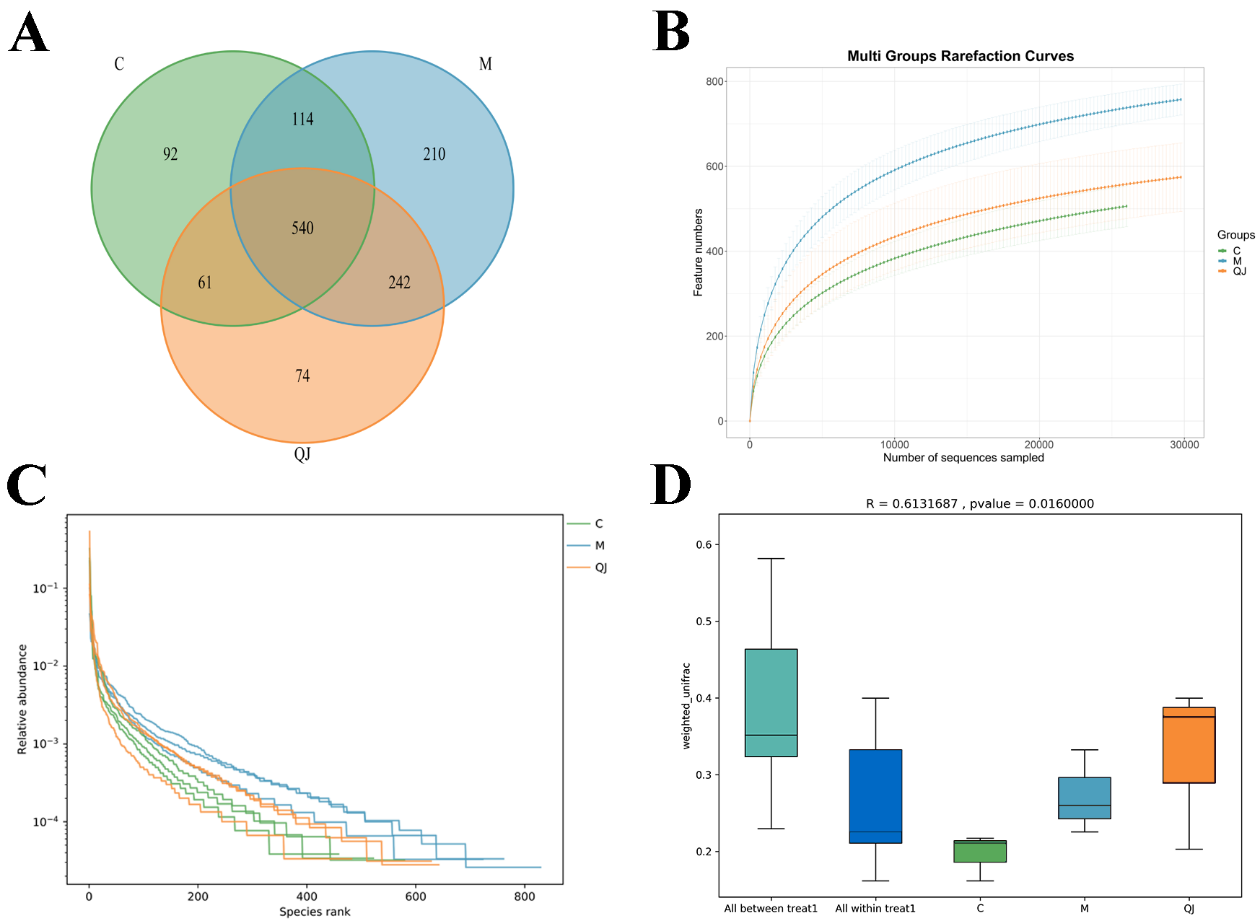

Figure S1. Supplementary figures of 16S rRNA analysis. (A) OTU Venn analysis. (B)Rarefaction curves. (C)Rank abundance curves. (D) Analysis of similarity (ANOSIM) based on weighted unifrac.

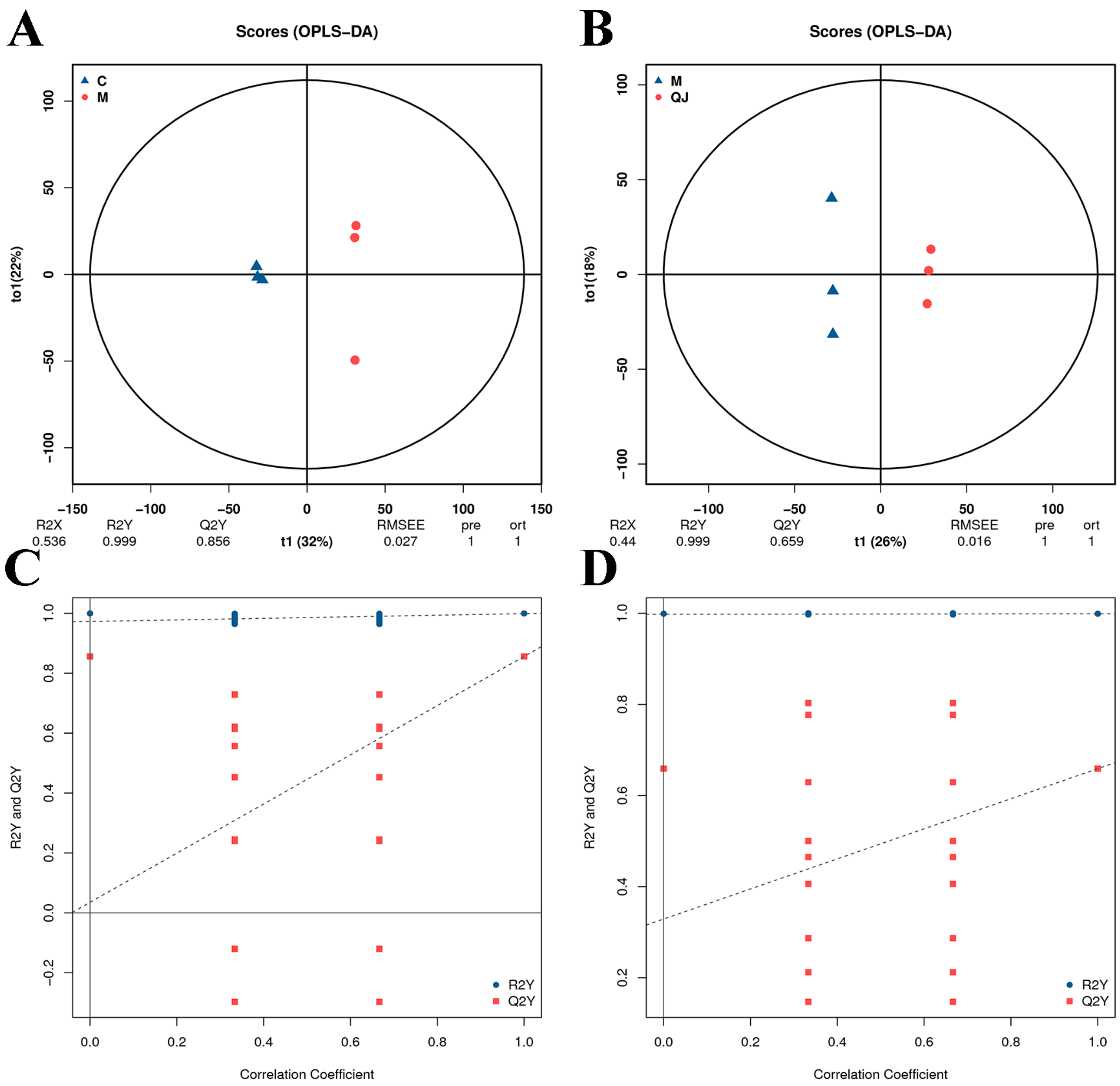

Figure S2. Orthogonal partial least squares discriminant analysis (OPLS-DA). (A, C) Score plots of OPLS-DA and the corresponding coefficient of loading plot between C and M groups. (B, D) Score plots of OPLS-DA and the corresponding coefficient of loading plot between M and QJ groups.

2 Supplementary Tables

Table S3. Co-differential metabolites in intestinal contents after QJ treatment

NO.	metabolite	Rt (min)	m/z	Formula	VIP		log2FC		Trend	
					C vs M	M vs QJ	C vs M	M vs QJ	C vs M	M vs QJ
1	(3S,3'R,4xi)-beta,beta-Carotene-3,3',4-triol	10.38	584.4267	C40H56O3	1.54	1.74	-0.82	0.73	↓*	↑*
2	2'-Hydroxyenterolactone	5.29	335.0856	C18H18O5	1.68	1.93	-3.45	2.85	↓*	↑**
3	(±)-Enterolactone	5.3	281.1186	C18H18O4	1.69	1.79	-2.49	1.84	↓*	↑*
4	7-Hydroxyenterolactone	4.36	315.1252	C18H18O5	1.65	1.63	-1.08	0.75	↓**	↑*
5	TR-Saponin C	4.94	1105.486	C54H82O21	1.66	1.87	1.67	-1.71	↑*	↓*
6	Aescin	4.5	1165.526	C55H86O24	1.73	1.85	0.96	-0.83	↑**	↓*
7	Soyasaponin A2	4.99	1107.549	C53H86O24	1.65	1.83	2.12	-2.05	↑*	↓*
8	28-Glucosyloleanolic acid 3-[rhamnosyl-(1->2)-galactosyl-(1->3)-glucuronide]	4.12	1151.503	C54H84O24	1.74	1.79	0.96	-0.72	↑**	↓*
9	Cinnamyl phenylacetate	5.14	297.1127	C17H16O2	1.63	1.77	-2.12	1.5	↓*	↑*
10	cis-Resveratrol 3-O-glucuronide	3.72	403.1056	C20H20O9	1.69	1.86	2.05	-1.98	↑*	↓*
11	Ampeloside Bf1	5.28	1159.556	C51H86O26	1.63	1.79	2.02	-1.83	↑*	↓*
12	Desglucocheirotoxin	5.14	1159.546	C29H42O10	1.69	1.87	1.98	-1.77	↑*	↓*
13	Physalolactone B	6.82	499.3035	C30H44O6	1.68	1.84	1.74	-1.6	↑*	↓*
14	Antibiotic X 14889A	9.98	584.4304	C33H60O8	1.63	1.74	-0.79	0.69	↓*	↑*
15	8-Acetyl-T2 tetrol	3.4	361.1267	C17H24O7	1.76	1.94	3.26	-3.04	↑**	↓**
16	7-Methylguanine	0.75	166.0697	C6H7N5O	1.6	1.72	0.73	-0.65	↑*	↓*
17	7,8-Dihydro-2'-deoxyguanosine	0.8	290.0855	C10H15N5O4	1.76	1.91	0.76	-0.47	↑**	↓**
18	Dexamethasone isonicotinate	4.34	496.2064	C28H32FNO6	1.64	1.87	1.08	-1.31	↑*	↓**
19	Aflatoxin B1 dialcohol	4	295.0982	C18H18O6	1.51	1.65	-1.53	1.53	↓*	↑*
20	Atropaldehyde	5.3	133.065	C9H8O	1.69	1.72	-2.07	1.52	↓*	↑*
21	Ganoderic acid L	9.57	535.3278	C30H46O8	1.66	1.7	0.75	-0.48	↑**	↓*
22	5-Hydroxy-3,3',4',7,8-pentamethoxyflavone	3.78	387.1089	C20H20O8	1.66	1.82	3.65	-3.09	↑*	↓*
23	2,3-Dihydroxypropyl octanoate	3.45	239.1256	C11H22O4	1.71	1.81	-2.53	1.79	↓**	↑*
24	(2E,4E)-2,4-Nonadien-1-ol	7.88	123.1154	C9H16O	1.55	1.84	0.47	-0.53	↑*	↓**
25	(E)-2-Methyl-2-buten-1-ol O-beta-D-Glucopyranoside	2.57	269.1011	C11H20O6	1.6	1.73	0.9	-0.96	↑*	↓*
26	7,8-Dihydrovomifoliol 9-[rhamnosyl-(1->6)-glucoside]	10.07	535.2731	C25H42O12	1.56	1.71	-0.99	1.45	↓*	↑*
27	17-Iodoheptadecanoic acid	5.5	837.304	C17H33IO2	1.66	1.85	3.2	-4.39	↑*	↓*
28	Pelargonic acid	3.21	123.1156	C9H18O2	1.7	1.67	1.84	-0.45	↑**	↓*
29	Talaromycin A	4.95	229.143	C12H22O4	1.73	1.72	-1.87	1.14	↓**	↑*
30	(2S,3R)-2-Acetamido-3-hydroxy-4-methylpentanoate	4.83	959.4883	C46H69N7O15	1.7	1.87	1.33	-0.99	↑*	↓*
31	6-Fluoromevalonate	4.46	131.0501	C6H9FO3	1.76	1.95	4.26	-3.73	↑**	↓**
32	2-Phenylethyl 3-phenyl-2-propenoate	5.3	253.1254	C17H16O2	1.67	1.63	-1.12	0.68	↓**	↑*
33	Trihydroxycoprostanoic acid	6.82	485.3232	C28H48O5	1.73	1.79	2.75	-1.43	↑*	↓*
34	Dihydrojasmone	7.84	149.1313	C11H18O	1.75	1.9	1.1	-0.86	↑**	↓**
35	Adhumulinic acid	4.19	287.1278	C15H22O4	1.72	1.81	-2.16	1.37	↓**	↑*

36	Neamine	3.97	305.185	C12H26N4O6	1.57	1.88	0.41	-0.65	↑*	↓**
37	7-Ketodeoxycholic acid	6.52	811.5306	C24H38O5	1.71	1.71	1.74	-0.69	↑*	↓*
38	p-Salicylic acid	2.95	137.0237	C7H6O3	1.73	1.8	-2.18	2.86	↓*	↑*
39	Glutamylisoleucine	5.95	299.0993	C11H20N2O5	1.76	1.94	3.92	-3.96	↑**	↓**
40	Hydroxyprolyl-Serine	3.45	239.0628	C8H14N2O5	1.72	1.89	1.19	-1.21	↑**	↓**
41	5-L-Glutamyl-taurine	2.21	275.0269	C7H14N2O6S	1.72	1.86	6.04	-2.93	↑*	↓**
42	Tolmetin glucuronide	4.32	451.1679	C21H23NO9	1.67	1.86	3.43	-3.57	↑*	↓*
43	Cyclosquamosin B	5.08	872.4487	C39H65N9O10S	1.52	1.83	0.48	-0.83	↑*	↓**
44	Cyclosquamosin D	4.73	881.4018	C41H56N8O11	1.71	1.89	2.68	-2.8	↑*	↓*
45	Leuphasyl	5.01	1161.568	C29H39N5O7	1.65	1.74	2.95	-1.87	↑*	↓*
46	Isoleucylproline	2.16	227.1372	C11H20N2O3	1.58	1.73	0.99	-0.99	↑*	↓*
47	(1R,2R,4R,5S)-(+)-p-Menthane-2,5-diol	9.59	139.1102	C9H18O3	1.68	1.84	2.44	-2.21	↑*	↓*
48	Terbutryn	9	283.1791	C10H19N5S	1.66	1.7	-0.22	0.22	↓**	↑*
49	31-Hydroxy rifabutin	5.1	885.4358	C46H62N4O12	1.77	1.95	2.28	-2.71	↑**	↓**
50	6H-Oxazaphosphinine	0.72	200.9991	C3H4NOP	1.77	1.9	1.61	-1.79	↑**	↓*
51	3-(5-Methyl-2-furanyl)butanal	3.64	151.0748	C9H12O2	1.74	1.88	3.84	-3.02	↑**	↓**
52	3-Hydroxy-6,7,8-trimethoxy-2-naphthoate	6.52	299.0517	C14H14O6	1.73	1.88	2.04	-1.62	↑**	↓**
53	Felbamate	4.96	283.0965	C11H14N2O4	1.61	1.86	-2.71	2.63	↓*	↑**
54	3-(2-Hydroxyphenyl)propionic acid	5.29	165.0543	C9H10O3	1.63	1.83	-3.94	3.44	↓*	↑*
55	Naproxcinod	4.32	368.114	C18H21NO6	1.61	1.82	2.96	-4.96	↑*	↓*
56	7-Ethyl-3,5-dimethyl-2E,4E,6E,8E-decatetraene	7.69	191.1762	C14H22	1.62	1.76	0.94	-0.85	↑*	↓*
57	3beta,6beta-Dihydroxynortropane	0.68	144.102	C7H13NO2	1.65	1.75	4.24	-2.14	↑*	↓*
58	Piquindone	4.04	267.155	C15H22N2O	1.55	1.74	-0.81	0.63	↓*	↑*
59	2-[(L-Alanin-3-ylcarbamoyl)methyl]-3-(2-aminoethylcarbamoyl)-2-hydroxypropanoate	5.16	341.1112	C11H20N4O7	1.75	1.74	3.9	-0.94	↑**	↓*
60	2-(1-Pentenyl)furan	5.89	137.0968	C9H12O	1.51	1.73	-1.01	1.75	↓*	↑*
61	5''-Phosphoribostamycin	4.02	573.1472	C17H35N4O13P	1.72	1.6	-2	1.37	↓**	↑*

C, M, QJ groups (n=3). Rt, Retention time; VIP, Variable important in projection; FC, fold-change. *, $P < 0.05$; **, $P < 0.01$.
